# Development and validation of a nomogram for predicting postoperative complications after pancreaticoduodenectomy: a retrospective cohort study

**DOI:** 10.3389/fonc.2026.1839358

**Published:** 2026-05-20

**Authors:** Tariq Azam, Haitham Salameen, Yun-Bing Wang, Wei-Long Du, Saed Woraikat, Xiu-Lin Wang, Wen-Feng Zhang, Jian-Ping Gong

**Affiliations:** 1 Department of Hepatobiliary Surgery, The Second Affiliated Hospital of Chongqing Medical University, Chongqing, China; 2 Centre for Lipid Research & Chongqing Key Laboratory of Metabolism on Lipid and Glucose, The Second Affiliated Hospital of Chongqing Medical University, Chongqing, China; 3 The Second Clinical College, Chongqing Medical University, Chongqing, China

**Keywords:** albumin, decision curve analysis, nomogram, NRS-2002, nutritional risk, pancreatic texture, pancreaticoduodenectomy, postoperative complications

## Abstract

**Background:**

Postoperative complications occur in 30–50% of patients undergoing pancreaticoduodenectomy and critically influence both recovery and oncological outcomes. Existing prediction models target pancreatic fistula in isolation, leaving the full spectrum of postoperative morbidity unaddressed. We developed and internally validated a nomogram to predict any clinically significant complication within 30 days of pancreaticoduodenectomy, integrating anatomical, pathological, surgical, and systemic perioperative variables.

**Methods:**

This single-centre retrospective cohort study enrolled consecutive adults undergoing pancreaticoduodenectomy between 2020 and 2024. The primary endpoint was any Clavien-Dindo grade II–IV complication within 30 days. Predictors were identified through a pre-specified hybrid scoring system combining univariate logistic regression, multivariable logistic regression, and LASSO penalised regression with 10-fold cross-validation; variables scoring above 3 were retained. Surgical approach was included as a pre-specified forced predictor. Internal validation employed 1000-iteration bootstrap resampling and 100-iteration repeated stratified cross-validation. Clinical utility was quantified by decision curve analysis.

**Results:**

Of 265 patients (mean age 62.0 years; 55.5% male), 97 (36.6%) developed Clavien-Dindo grade II–IV complications. The nomogram incorporated six data-driven predictors — pancreatic texture, histological diagnosis, preoperative albumin, CRP, anatomical site, and NRS-2002 nutritional score — with surgical approach as a forced inclusion. Pancreatic texture was the dominant predictor (OR 3.49, 95% CI 1.71–7.11; P<0.001); preoperative hypoalbuminaemia independently conferred excess risk (OR 0.93 per g/L; P = 0.016). The model achieved an apparent AUC of 0.747 (bootstrap 95% CI 0.704–0.825), an optimism-corrected AUC of 0.704, and satisfactory calibration (Hosmer–Lemeshow P = 0.686). Repeated cross-validation yielded a mean validation AUC of 0.707 ± 0.063. Decision curve analysis confirmed net benefit across threshold probabilities of 8–87%.

**Conclusions:**

This internally validated nomogram — incorporating pancreatic texture, histological diagnosis, anatomical site, CRP, surgical approach, preoperative albumin, and NRS-2002 — provides clinically actionable perioperative risk stratification for significant complications following pancreaticoduodenectomy, pending prospective external validation. The convergent data-driven selection of two complementary nutritional predictors positions preoperative nutritional optimisation as a modifiable target for complication reduction in this high-risk population.

## Introduction

Pancreaticoduodenectomy remains the only potentially curative procedure for pancreatic head and periampullary malignancies, yet it carries one of the highest morbidity rates in abdominal surgery. Postoperative complication rates of 30–50% are consistently reported in large prospective series and multicentre registries, encompassing pancreatic fistula, bile leak, delayed gastric emptying, intra-abdominal abscess, intestinal fistula, and postoperative hemorrhage (1–3). Beyond the immediate clinical impact, these complications carry significant oncological consequences: adjuvant chemotherapy — which prolongs survival by 5–10 months in randomised trials (4, 5) is omitted in over 30% of patients who experience major postoperative morbidity, compromising long-term outcomes in a disease where every therapeutic opportunity matters (6). Accurate perioperative identification of patients at highest risk is therefore a clinical priority.

Risk prediction in pancreaticoduodenectomy has been dominated by models targeting postoperative pancreatic fistula specifically. The Fistula Risk Score and its derivatives incorporate pancreatic texture, main pancreatic duct diameter, intraoperative blood loss, and pathological diagnosis, achieving AUCs of 0.64–0.80 in external validation cohorts (7, 8). More than 60 fistula-specific prediction models have been published to date (9, 10). Yet pancreatic fistula accounts for fewer than half of all complicated patients; bile leak, intra-abdominal abscess, intestinal fistula, and delayed gastric emptying collectively represent the majority of the postoperative morbidity burden. A model predicting the full spectrum of clinically significant complications — defined by Clavien-Dindo grade II–IV — addresses a gap that fistula-specific instruments cannot fill. Notably, published CT-based or radiomics-based approaches in this field have similarly focused on fistula-specific endpoints and have not been externally validated for composite Clavien-Dindo morbidity; the present work targets this distinct endpoint using routinely available clinical variables.

A fundamental limitation of existing models is their near-exclusive focus on local anatomical parameters, particularly pancreatic texture and ductal morphology, at the expense of systemic physiological determinants of surgical risk. Preoperative nutritional status is among the most clinically relevant and potentially modifiable of these determinants, yet it is rarely incorporated as a formal predictor in pancreatic resection models. The pathophysiological basis for this association is well-established: protein-energy depletion impairs anastomotic healing through reduced collagen synthesis and impaired fibroblast proliferation; hypoalbuminaemia reflects the biochemical consequence of this deficit through diminished hepatic synthetic output; and systemic inflammation, indexed by CRP, suppresses albumin as its inverse acute-phase counterpart, creating a converging nutritional-inflammatory axis of vulnerability (11, 12). Patients undergoing pancreaticoduodenectomy are particularly susceptible to preoperative nutritional depletion, given the combined effects of tumour-related malabsorption, biliary obstruction, anorexia, and cancer cachexia, resulting in rates of clinically significant nutritional risk exceeding 70% in some series (13, 14).

Nutritional risk screening tools, of which the Nutritional Risk Screening 2002 (NRS-2002) is the most widely validated and ESPEN-endorsed instrument, capture a composite clinical dimension of nutritional risk that complements biochemical markers such as albumin (14–16). While albumin reflects the biochemical consequence of nutritional depletion at the level of hepatic synthetic function, NRS-2002 integrates disease severity and functional nutritional impairment, identifying patients whose nutritional requirements are elevated beyond what their current intake can meet. Together, these two predictors represent complementary dimensions of the same underlying physiological vulnerability — one objective and biochemical, one clinical and functional — providing a more complete nutritional risk assessment than either alone.

We therefore aimed to develop and internally validate a nomogram for predicting any Clavien-Dindo grade II–IV complication within 30 days of pancreaticoduodenectomy, incorporating a broad range of preoperative, intraoperative, and pathological candidate predictors identified through a pre-specified multi-method variable selection framework. Surgical approach was incorporated as a forced predictor given prior evidence of differential complication profiles between open and minimally invasive pancreaticoduodenectomy (17) and the predominantly minimally invasive practice at this centre. Clinical utility was assessed by decision curve analysis, and complication-specific discriminative performance was examined as a secondary objective.

## Methods

### Study design and setting

This retrospective observational cohort study was conducted at the Department of Hepatobiliary Surgery, The Second Affiliated Hospital of Chongqing Medical University, Chongqing, China, from January 2020 through December 2024. The centre performs the majority of pancreaticoduodenectomies via minimally invasive approaches (laparoscopic or robotic), reflecting institutional case-volume and technical expertise. Institutional ethics committee approval was obtained and all procedures adhered to the principles of the Declaration of Helsinki. Reporting follows the TRIPOD (Transparent Reporting of a Multivariable Prediction Model for Individual Prognosis or Diagnosis) statement; a completed TRIPOD checklist is provided as a **Supplementary File**.

### Participants

**Figure 1:**
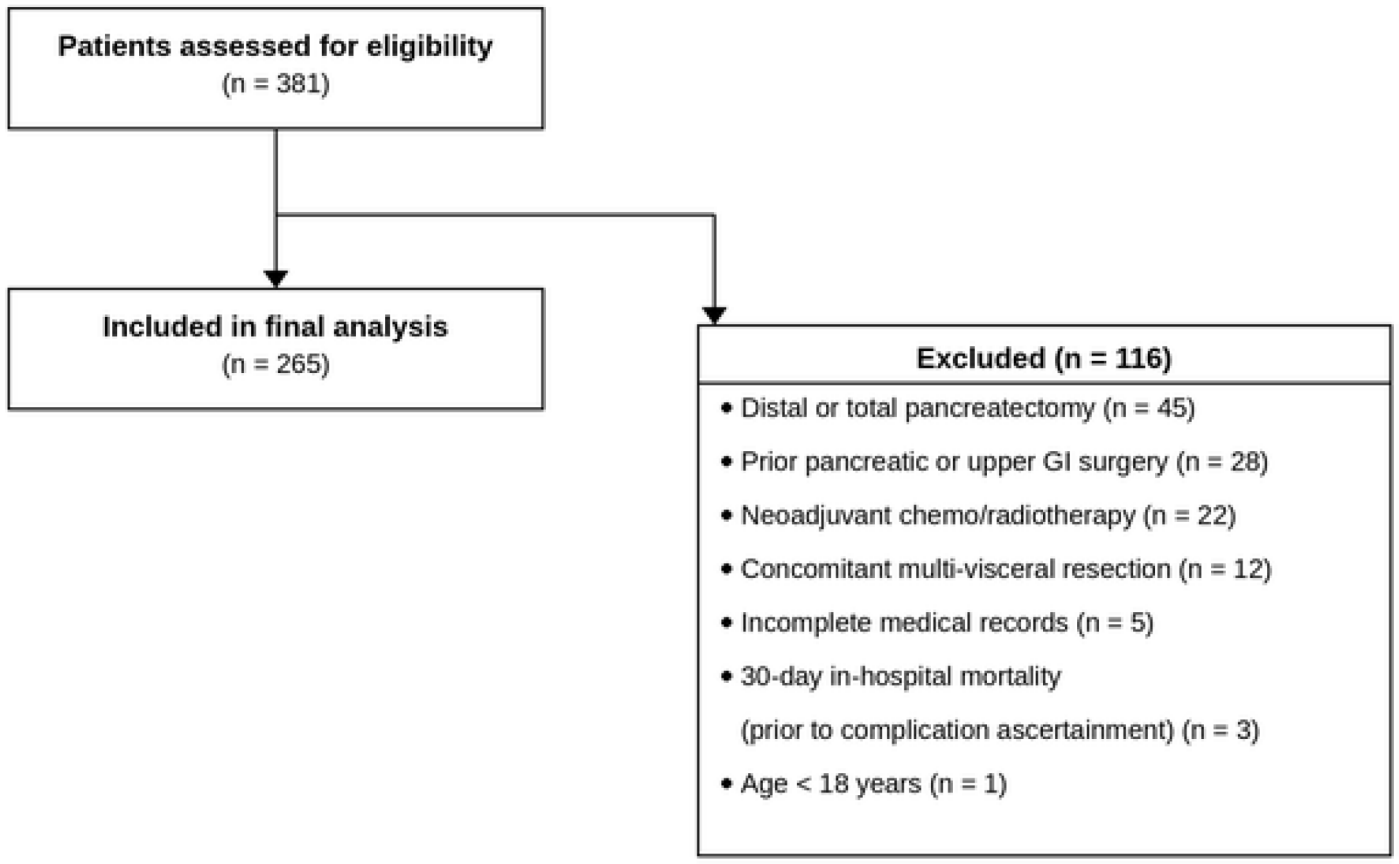
Flow diagram of patient selection. A total of 381 consecutive patients undergoing pancreaticoduodenectomy at the Department of Hepatobiliary Surgery, The Second Affiliated Hospital of Chongqing Medical University (2020–2024) were assessed for eligibility. After applying exclusion criteria, 116 patients were excluded and 265 were included in the final analysis.

All consecutive adult patients (aged ≥18 years) undergoing pancreaticoduodenectomy for malignant or benign pancreatic or periampullary pathology during the study period were eligible. Of 381 patients assessed for eligibility, 116 were excluded: 45 had undergone distal or total pancreatectomy rather than pancreaticoduodenectomy; 28 had a history of prior pancreatic or upper gastrointestinal surgery; 22 had received neoadjuvant chemotherapy or radiotherapy prior to surgery; 12 underwent concomitant multi-visceral resection not directly related to the primary pathology; 5 had incomplete medical records precluding complete case analysis; 3 died within 30 days of surgery and were therefore outside the scope of the Clavien-Dindo grade II–IV morbidity endpoint — Grade V (death) constitutes a categorically distinct outcome within the Clavien-Dindo classification and was not the subject of this study; and 1 patient was aged under 18 years. The remaining 265 patients formed the final study cohort and were included in all analyses (**Figure 1**).

### Data collection and predictors

Data were extracted from electronic health records using a pre-specified case report form. Baseline characteristics included age, sex, BMI, and comorbidities including diabetes mellitus and hypertension. Nutritional risk was assessed using the NRS-2002, a validated composite screening tool integrating impairment of nutritional status and disease-related increase in requirements; a score of 3 or higher indicates clinically significant risk requiring nutritional intervention (14). Preoperative laboratory investigations obtained within 72 hours before surgery included serum CRP (mg/L), albumin (g/L), red blood cell count (×10¹²/L), alanine aminotransferase (U/L), aspartate aminotransferase (U/L), and total bilirubin (µmol/L).

Intraoperative variables included pancreatic texture — classified as Soft (friable, non-fibrotic parenchyma with reduced suture-holding capacity) or Firm/Hard (fibrotic parenchyma with adequate suture-holding capacity and reduced exocrine secretion) using a pre-specified binary classification. This binary scheme was chosen in preference to the conventional three-level grading on the basis that Firm and Hard textures share the same mechanistically relevant property — parenchymal fibrosis — while the distinction between them lacks standardised criteria and is susceptible to inter-surgeon variability. Additional intraoperative variables included surgical approach (open versus minimally invasive surgery [MIS]: laparoscopic or robotic), operative duration, estimated blood loss, and intraoperative transfusion. Histological diagnosis and anatomical site were obtained from final pathology reports.

### Outcome measures

The primary endpoint was any Clavien-Dindo grade II–IV complication within 30 days of surgery, chosen to capture the full clinically meaningful spectrum of postoperative morbidity. Patients who died within 30 days were outside the scope of this morbidity endpoint: the Clavien-Dindo grading system characterises therapeutic responses in surviving patients across Grades I–IV, while Grade V (death) constitutes a categorically distinct outcome (18). Secondary endpoints included clinically relevant postoperative pancreatic fistula (CR-POPF; ISGPS grade B or C) (19, 20), delayed gastric emptying (ISGPS grade B or C), bile leak, intra-abdominal abscess, and intestinal fistula, each defined per established international consensus criteria (19, 21–23). All complications were prospectively recorded in a dedicated database and retrospectively validated by two independent reviewers; discordances were resolved by a third reviewer.

### Statistical analysis

All analyses were performed in R (version 4.5.0). Candidate predictors with more than 40% missing data were excluded prior to analysis (**Supplementary Table 6** provides the complete missing data profile for all 33 candidate variables; all predictors retained in the final model had ≤1.1% missing data, justifying complete-case analysis throughout). Complete case analysis was performed throughout. Owing to the retrospective design, outcome ascertainment was performed by chart review without formal blinding to predictor values. Variance inflation factor (VIF) screening was applied to detect multicollinearity (GVIF^(1/2df) threshold 10) before variable selection.

Variable selection employed a pre-specified three-component hybrid scoring system designed to balance stability and clinical plausibility: (1) univariate logistic regression — scored up to 3 points (P<0.05 = 2, P<0.10 = 1.5, P<0.20 = 1, P<0.30 = 0.5; plus 1 point for OR >2.0 or <0.5); (2) fully adjusted multivariable logistic regression incorporating all approximately 20 candidate variables — scored up to 6 points (P<0.05 = 5, P<0.10 = 4.5, P<0.20 = 3, P<0.30 = 1; plus 1 point for OR >2.0 or <0.5); (3) LASSO penalized regression (24) with 10-fold cross-validation (glmnet package) — 3 points for retention at lambda.1se, 1 point at lambda.min only. Variables with a composite score strictly exceeding 3 were retained by data-driven selection. This multi-source consensus approach reduces both the probability of retaining noise variables and the probability of excluding clinically relevant predictors in a moderate-sized sample. Surgical approach was additionally included as a forced predictor, independent of its composite score, on two pre-specified grounds: (i) published randomised and prospective evidence demonstrating differential complication profiles between open and minimally invasive pancreaticoduodenectomy (17), and (ii) the predominantly MIS practice at this centre (69.4%), which creates a case-mix context in which approach constitutes a clinically relevant source of outcome heterogeneity requiring adjustment regardless of its statistical signal within this specific dataset.

The final model was fitted by multivariable logistic regression. Discrimination was quantified by the AUC (pROC package) with 1000-iteration bootstrap resampling to estimate optimism and produce an optimism-corrected AUC. Separately, 100-iteration repeated stratified 80:20 cross-validation was performed, yielding mean ± SD validation AUC. Calibration was assessed by the Hosmer–Lemeshow test (g=8 groups) with calibration plots. A nomogram was constructed using the rms package. Clinical utility was evaluated by decision curve analysis (dcurves package) across threshold probabilities of 5–90%. Subgroup analyses were pre-specified for surgical approach, nutritional risk (NRS-2002 ≥3 versus <3), age (≥65 versus <65 years), and sex, with likelihood ratio interaction tests. A sensitivity analysis comparing the hybrid selection model against LASSO lambda.1se alone was performed (**Supplementary Table 4**).

## Results

### Cohort characteristics and complication incidence

Between January 2020 and December 2024, 265 consecutive patients underwent pancreaticoduodenectomy and met all eligibility criteria. The cohort comprised 147 men (55.5%) and 118 women (44.5%), with a mean age of 62.0 ± 11.4 years and mean BMI of 22.6 ± 3.2 kg/m². Procedures were performed via MIS in 184 patients (69.4%) and via open approach in 81 (30.6%), reflecting this centre’s predominantly minimally invasive practice. Pancreatic texture was Soft in 50 patients (18.9%) and Firm/Hard in 215 (81.1%). The cohort carried a high burden of preoperative nutritional risk, with clinically significant NRS-2002 scores (≥3) present in 191 patients (72.1%), consistent with the nutritional vulnerability characteristic of pancreatic and periampullary malignancy populations. The mean preoperative albumin was within the normal range overall but was significantly lower in the complication group (35.79 versus 37.93 g/L; P = 0.001). Histologically, carcinoma was the most frequent diagnosis (184, 69.4%), followed by non-neoplastic disease (40, 15.1%), benign lesions (21, 7.9%), precursor lesions (12, 4.5%), and neuroendocrine tumours (8, 3.0%). Full baseline characteristics are presented in **Table 1**.

**Table 1:** Baseline characteristics by complication status.

Variable	Statistic	No complication (n=168)	Complication (n=97)	*P*-value	Test
*Age (years)*	mean (SD)	61.99 (11.36)	62.16 (11.64)	0.908	t-test
*Sex*	Male n (%)	92 (54.8%)	55 (56.7%)	0.859	Chi-sq
	Female n (%)	76 (45.2%)	42 (43.3%)		
*BMI (kg/m²)*	mean (SD)	22.58 (3.10)	22.71 (3.36)	0.760	t-test
*NRS-2002 score*	median (IQR)	3 (2–4)	3 (2–4)	0.574	Mann–Whitney
*Diabetes mellitus*	No n (%)	149 (88.7%)	81 (83.5%)	0.311	Chi-sq
	Yes n (%)	19 (11.3%)	16 (16.5%)		
*Hypertension*	No n (%)	128 (76.2%)	71 (73.2%)	0.692	Chi-sq
	Yes n (%)	40 (23.8%)	26 (26.8%)		
*Pancreatic texture†*	Firm/Hard n (%)	149 (88.7%)	66 (68.0%)	<0.001	Chi-sq
	Soft n (%)	19 (11.3%)	31 (32.0%)		
*Surgical approach*	MIS n (%)	118 (70.2%)	66 (68.0%)	0.814	Chi-sq
	Open n (%)	50 (29.8%)	31 (32.0%)		
*Operative duration (min)*	mean (SD)	447.61 (114.44)	433.16 (118.38)	0.340	t-test
*Blood loss (mL)*	median (IQR)	200 (200–400)	200 (200–400)	0.986	Mann–Whitney
*Histological diagnosis*	Carcinoma n (%)	127 (75.6%)	57 (58.8%)	0.014	Chi-sq
	NET n (%)	4 (2.4%)	4 (4.1%)		
	Precursor n (%)	8 (4.8%)	4 (4.1%)		
	Benign n (%)	7 (4.2%)	14 (14.4%)		
	Non-neoplastic n (%)	22 (13.1%)	18 (18.6%)		
*Anatomical site*	Pancreas n (%)	98 (58.3%)	65 (67.0%)	0.127	Chi-sq
	Ampulla n (%)	42 (25.0%)	14 (14.4%)		
	Duodenum n (%)	28 (16.7%)	18 (18.6%)		
*Albumin (g/L)*	mean (SD)	37.93 (5.31)	35.79 (5.00)	0.001	t-test
*RBC (×10¹²/L)*	mean (SD)	3.70 (0.61)	3.63 (0.60)	0.418	t-test
*CRP (mg/L)*	median (IQR)	13.69 (5.00–45.10)	18.69 (5.00–76.56)	0.170	Mann–Whitney
*ALT (U/L)*	median (IQR)	121.0 (61.0–274.0)	135.0 (70.0–253.0)	0.751	Mann–Whitney
*AST (U/L)*	median (IQR)	91.0 (56.0–180.8)	97.0 (60.0–154.0)	0.909	Mann–Whitney
*Total bilirubin (µmol/L)*	median (IQR)	114.3 (36.6–175.2)	100.3 (45.6–182.4)	0.864	Mann–Whitney

BMI, body mass index; NRS-2002, Nutritional Risk Screening 2002; MIS, minimally invasive surgery; CRP, C-reactive protein; RBC, red blood cell count; ALT, alanine aminotransferase; AST, aspartate aminotransferase. Continuous variables: mean (SD) when normally distributed, median (IQR) when skewed. Between-group comparisons: t-test (normal continuous), Mann–Whitney U (skewed), chi-squared (categorical), Fisher exact (cells <5). †Original three-level texture: no-complication group — Firm 129 (76.8%), Hard 20 (11.9%), Soft 19 (11.3%); complication group — Firm 46 (47.4%), Hard 20 (20.6%), Soft 31 (32.0%). Displayed as binary (Firm/Hard vs Soft) as used in the final model.

Within 30 days of surgery, 97 patients (36.6%) developed Clavien-Dindo grade II–IV complications (**Table 2**). Pancreatic fistula was the most frequent individual complication (39, 14.7%), followed by bile leak (26, 9.8%), intra-abdominal abscess (17, 6.4%), intestinal fistula (14, 5.3%), and delayed gastric emptying (11, 4.2%).

**Table 2:** Postoperative complication breakdown (N = 265).

Complication	n	% (N = 265)
*Pancreatic fistula (CR-POPF; ISGPS grade B/C)*	39	14.7
*Bile leak*	26	9.8
*Intra-abdominal abscess*	17	6.4
*Intestinal fistula*	14	5.3
*Delayed gastric emptying (ISGPS grade B/C)*	11	4.2
***Any complication (Clavien-Dindo grade II–IV)***	**97**	**36.6**

Individual complication categories are not mutually exclusive. CR-POPF, clinically relevant postoperative pancreatic fistula; ISGPS, International Study Group of Pancreatic Surgery. Bold row denotes the primary composite outcome (Any complication, Clavien-Dindo grade II-IV).

### Comparative analysis by complication status

Patients who developed complications were broadly similar to uncomplicated patients in demographic and operative characteristics, with no significant differences in age (P = 0.908), sex (P = 0.859), BMI (P = 0.760), operative duration (P = 0.340), blood loss (P = 0.986), or surgical approach (P = 0.814; **Table 1**). The absence of a significant difference in surgical approach is consistent with the balanced complication rates between MIS and open procedures in this predominantly MIS cohort. Two variables showed strong between-group differences: Soft pancreatic texture was markedly more prevalent among complicated patients (32.0% versus 11.3%; P<0.001), and serum albumin was significantly lower in the complication group (35.79 versus 37.93 g/L; P = 0.001). Histological diagnosis differed significantly between groups (P = 0.014), with benign pathology accounting for 14.4% of complicated patients versus 4.2% of uncomplicated patients. NRS-2002 scores and CRP did not differ significantly between groups (P = 0.574 and P = 0.170, respectively), though the consistent directional trends informed their variable selection signals.

### Univariate analysis

Univariate logistic regression identified pancreatic texture (OR 3.683, 95% CI 1.941–6.988; P<0.001) and preoperative albumin (OR 0.921 per g/L; P = 0.002) as the strongest predictors among continuous and binary variables (**Table 3**). Histological diagnosis showed a significant overall association (P = 0.016), driven predominantly by the benign category (OR 4.456 versus Carcinoma; P = 0.002). CRP was independently associated with complications (OR 1.006 per mg/L; P = 0.029). Ampullary anatomical site was protective on univariate analysis (OR 0.503; P = 0.048). NRS-2002 score did not reach univariate significance (P = 0.484; univariate score 0.0), but its directional multivariable signal (P = 0.146; scoring 3.0 points) and retention at lambda.min in LASSO (scoring 1.0 point) yielded a composite score of 4.0, strictly exceeding the retention threshold of 3. Surgical approach was not univariately associated with complications (P = 0.709), consistent with the balanced approach distribution in this cohort.

**Table 3:** Univariate logistic regression — all candidate predictors.

Variable	OR (95% CI)	*P* (level)	*P* (overall)
*Pancreatic texture: soft vs firm/hard*	3.683 (1.941–6.988)	**<0.001**	**<0.001**
*Preop albumin (per g/L)*	0.921 (0.875–0.970)	**0.002**	**0.002**
***Histological diagnosis (overall)***	—	—	**0.016**
*Benign vs carcinoma*	4.456 (1.707–11.634)	**0.002**	
*Non-neoplastic vs carcinoma*	1.823 (0.908–3.660)	0.091	
*NET vs carcinoma*	2.228 (0.538–9.224)	0.269	
*Precursor vs carcinoma*	1.114 (0.322–3.851)	0.865	
*Preop CRP (per mg/L)*	1.006 (1.001–1.011)	**0.029**	**0.029**
***Anatomical site (overall)***	—	—	0.116
*Ampulla vs pancreas*	0.503 (0.254–0.993)	**0.048**	
*Duodenum vs pancreas*	0.969 (0.496–1.894)	0.927	
*NRS-2002 score (per point)*	1.067 (0.889–1.281)	0.484	0.484
*Surgical approach: open vs MIS*	1.108 (0.646–1.902)	0.709	0.709
*Diabetes mellitus (yes vs no)*	1.549 (0.756–3.176)	0.232	0.232
*Preop RBC (per ×10¹²/L)*	0.843 (0.558–1.274)	0.418	0.418
*Hypertension (yes vs no)*	1.172 (0.661–2.078)	0.587	0.587
*BMI (per kg/m²)*	1.013 (0.936–1.095)	0.754	0.754
*Sex: female vs male*	0.924 (0.559–1.530)	0.760	0.760
*Age (per year)*	1.001 (0.980–1.023)	0.907	0.907
*Preop ALT (per U/L)*	1.000 (0.998–1.001)	0.642	0.642
*Preop AST (per U/L)*	1.000 (0.997–1.002)	0.705	0.705
*Total bilirubin (per µmol/L)*	1.000 (0.998–1.003)	0.835	0.835
*Blood loss (per mL)*	1.000 (0.999–1.002)	0.400	0.400
*Operative duration (per min)*	0.999 (0.997–1.001)	0.335	0.335

OR, odds ratio; CI, confidence interval; MIS, minimally invasive surgery; CRP, C-reactive protein; RBC, red blood cell count; NET, neuroendocrine tumour; NRS-2002, Nutritional Risk Screening 2002. P (overall) = likelihood ratio test across all levels for categorical variables. Bold values indicate statistical significance (P < 0.05).

### Variable selection

The composite scoring system identified six data-driven predictors meeting the retention threshold (composite score >3): pancreatic texture and histological diagnosis (12.0 each), albumin and CRP (10.0 each), anatomical site (7.0), and NRS-2002 score (4.0). Surgical approach did not meet the data-driven threshold (composite score 0.0) but was included as a pre-specified forced predictor for the reasons described in the Methods. Variables excluded from the final model included diabetes mellitus, hypertension, BMI, RBC, and all remaining candidates. Full scoring details are presented in **Table 4**.

**Table 4:** Composite variable selection scoring system.

Variable	Uni P	Uni OR	Uni Sc.	Multi P*	Multi OR*	Multi Sc.*	LASSO	Total score
Data-driven selected variables (composite score >3)								
*Texture (soft vs F/H)*	<0.001	3.683	3.0	0.001	3.773	6.0	3	**12.0**
*Histology*	0.016	4.456	3.0	0.003	5.767	6.0	3	**12.0**
*Preop albumin*	0.002	0.921	2.0	0.002	0.906	5.0	3	**10.0**
*Preop CRP*	0.029	1.006	2.0	0.012	1.008	5.0	3	**10.0**
*Anatomical site*	0.116	0.503	1.0	0.069	0.479	5.0	1	**7.0**
*NRS-2002 score*	0.484	1.067	0.0	0.146	1.186	3.0	1	**4.0**
Forced inclusion — pre-specified clinical justification								
*Surgical approach †*	0.709	1.108	0.0	0.338	0.718	0.0	0	0.0 (forced)
Excluded — composite score ≤3								
*Diabetes mellitus*	0.232	1.549	0.5	0.319	1.549	0.5	1	2.0
*Hypertension*	0.587	1.172	0.0	0.444	1.312	0.0	1	1.0
*BMI*	0.754	1.013	0.0	0.211	1.070	1.0	0	1.0
*Preop RBC*	0.419	0.843	0.0	0.951	1.017	0.0	0	0.0
*Surgery duration*	0.335	0.999	0.0	0.334	0.998	0.0	0	0.0
*Blood loss*	0.400	1.000	0.0	0.691	1.000	0.0	0	0.0
*Transfusion*	0.418	1.375	0.0	0.777	0.855	0.0	0	0.0
*ALT*	0.642	1.000	0.0	0.700	1.001	0.0	0	0.0
*AST*	0.705	1.000	0.0	0.782	0.999	0.0	0	0.0
*Sex*	0.760	0.924	0.0	0.739	0.897	0.0	0	0.0
*Total bilirubin*	0.835	1.000	0.0	0.636	1.001	0.0	0	0.0
*Age*	0.907	1.001	0.0	0.303	1.016	0.0	0	0.0

Scoring — Univariate: P<0.05 = 2pts, P<0.10 = 1.5pts, P<0.20 = 1pt, P<0.30 = 0.5pts; +1pt for OR>2.0 or <0.5. Multivariable: P<0.05 = 5pts, P<0.10 = 4.5pts, P<0.20 = 3pts, P<0.30 = 1pt; +1pt for OR>2.0 or <0.5. LASSO: 3pts at lambda.1se, 1pt at lambda.min only. Retention threshold: composite score STRICTLY >3 (score of exactly 3 excluded). †Surgical approach: score 0.0, included as pre-specified forced predictor. *Multi P and Multi OR derived from the fully adjusted candidate-pool model — NOT from the final fitted model. F/H, Firm/Hard; CRP, C-reactive protein; NRS-2002, Nutritional Risk Screening 2002. Bold values indicate statistical significance (P < 0.05).

### Final model and multivariable analysis

The seven-predictor model is presented in **Table 5**. Pancreatic texture remained the dominant predictor in multivariable analysis (OR 3.490, 95% CI 1.713–7.111; P<0.001), with Soft texture conferring a 3.5-fold increase in the odds of any clinically significant complication. Benign histological diagnosis was independently significant (OR 4.734, 95% CI 1.645–13.627; P = 0.004), a finding with biological plausibility given the strong association between benign pancreatic pathology and soft, non-fibrotic parenchyma. Preoperative albumin was independently and significantly associated with complications in the adjusted model (OR 0.925 per g/L; P = 0.016), confirming that nutritional depletion contributes to postoperative risk independent of texture and pathological characteristics. CRP attenuated toward non-significance (P = 0.204) in the presence of albumin, reflecting their shared inflammatory-nutritional axis — as CRP rises in states of systemic inflammation, albumin falls as its inverse acute-phase counterpart — while both were retained to preserve the full signal from opposing directions. NRS-2002 score retained a consistent directional effect (OR 1.165 per point; P = 0.152), contributing to model selection through its multivariable and LASSO evidence. Surgical approach showed OR 0.740 for open versus MIS (P = 0.364), with a direction consistent with published outcomes data from predominantly MIS centres.

**Table 5:** Multivariable logistic regression — final nomogram model.

Variable	OR (95% CI)	*P*-value
***Pancreatic texture: soft (vs firm/hard)***	**3.490 (1.713–7.111)**	**<0.001**
Histological diagnosis		
*Benign (vs carcinoma)*	**4.734 (1.645–13.627)**	**0.004**
*NET (vs carcinoma)*	2.845 (0.611–13.247)	0.183
*Precursor (vs carcinoma)*	0.941 (0.242–3.663)	0.930
*Non-neoplastic (vs carcinoma)*	1.489 (0.670–3.311)	0.329
*Preop albumin (per g/L)*	**0.925 (0.869–0.986)**	**0.016**
*Preop CRP (per mg/L)*	1.005 (0.997–1.013)	0.204
Anatomical site		
*Ampulla (vs pancreas)*	0.545 (0.256–1.162)	0.116
*Duodenum (vs pancreas)*	0.710 (0.328–1.537)	0.384
*NRS-2002 score (per point)*	1.165 (0.946–1.434)	0.152
*Surgical approach: open (vs MIS) ‡*	0.740 (0.386–1.417)	0.364

OR, odds ratio; CI, confidence interval; MIS, minimally invasive surgery; CRP, C-reactive protein; NET, neuroendocrine tumour; NRS-2002, Nutritional Risk Screening 2002. ‡Surgical approach included as forced predictor on pre-specified clinical grounds (see Methods). P-values differ from **Table 4** multivariable column, which was derived from the fully adjusted candidate-pool model incorporating all ~20 candidates before selection. Bold values indicate statistical significance (P < 0.05).

### Model performance and internal validation

**Figure 2:**
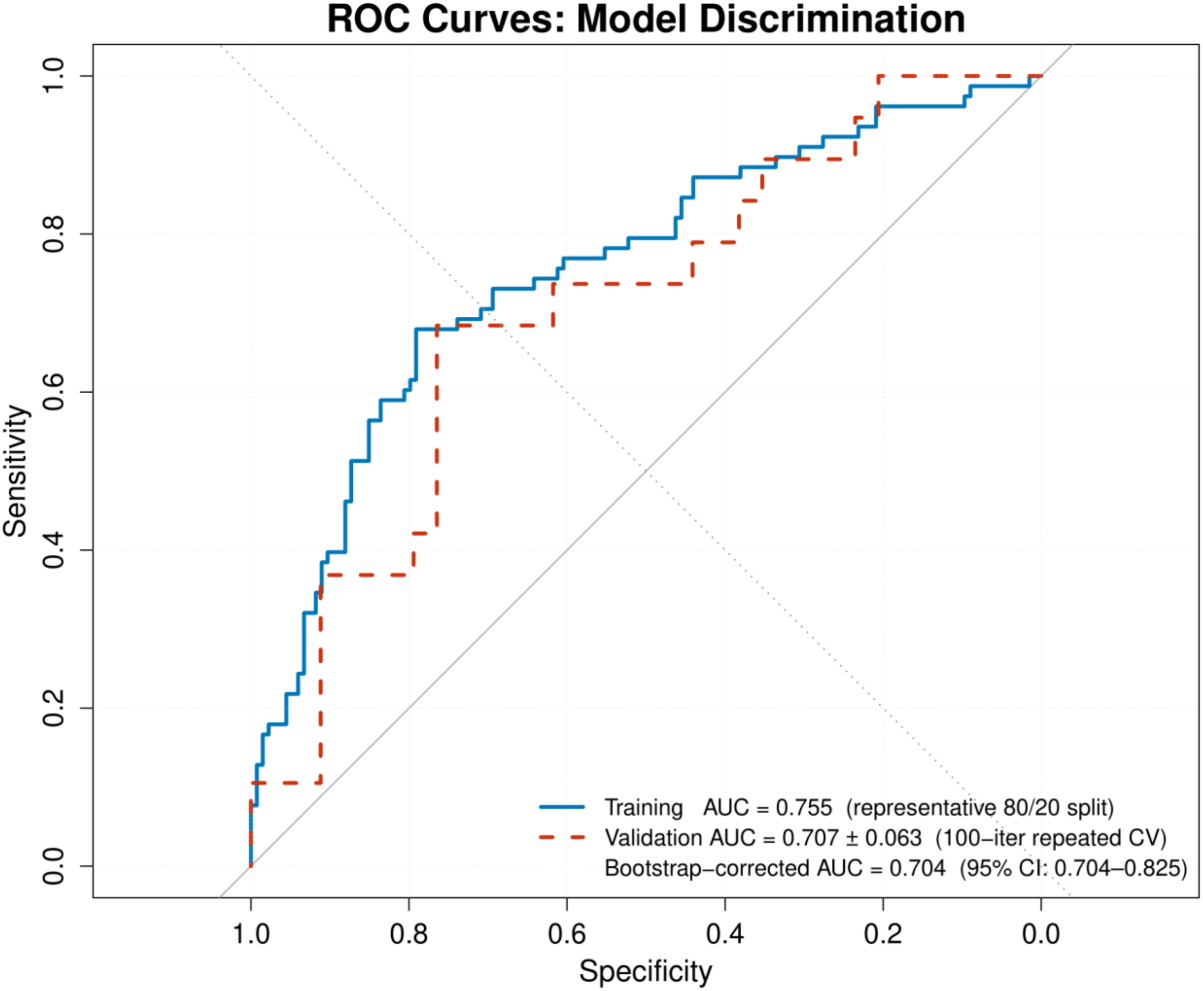
Receiver operating characteristic (ROC) curves for the prediction model. The solid curve represents a representative 80:20 training split (training AUC 0.755). The full-sample apparent AUC is 0.747 (bootstrap 95% CI 0.704–0.825). The dashed curve represents mean validation performance from 100-iteration repeated stratified cross-validation (mean AUC 0.707 ± 0.063; 95% CI 0.597–0.841). The optimism-corrected AUC of 0.704 is a separate bias-corrected point estimate derived from 1000-iteration bootstrap resampling and does not carry the same CI as the apparent AUC. The dotted diagonal represents chance discrimination.

**Figure 3:**
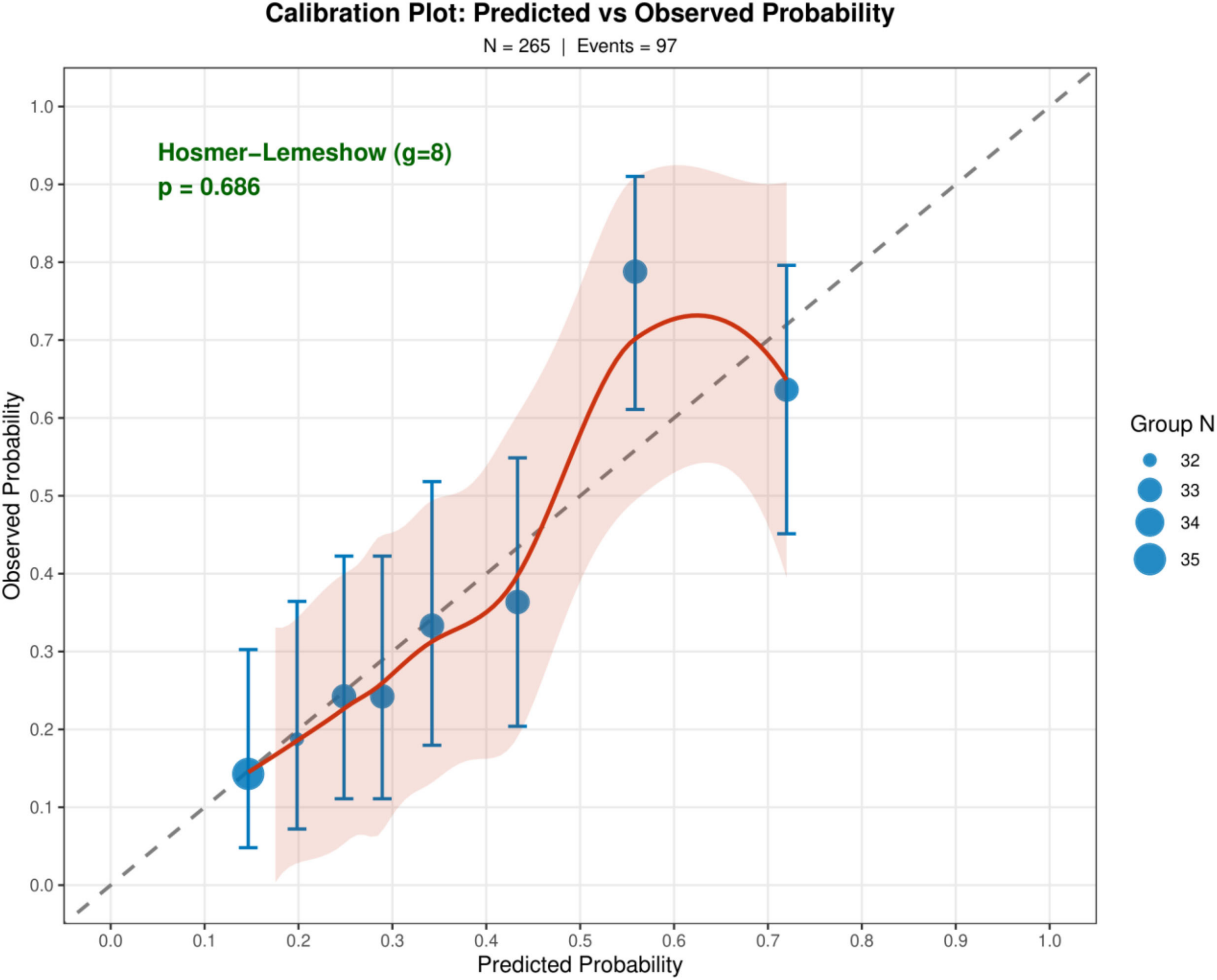
Calibration plot of the prediction model. Observed complication rates are plotted against model-predicted probabilities across eight risk groups. Points represent group means with 95% confidence intervals; bubble size is proportional to group n. The dashed 45° line represents perfect calibration. Hosmer–Lemeshow P = 0.686 (N = 265; events=97).

**Figure 4:**
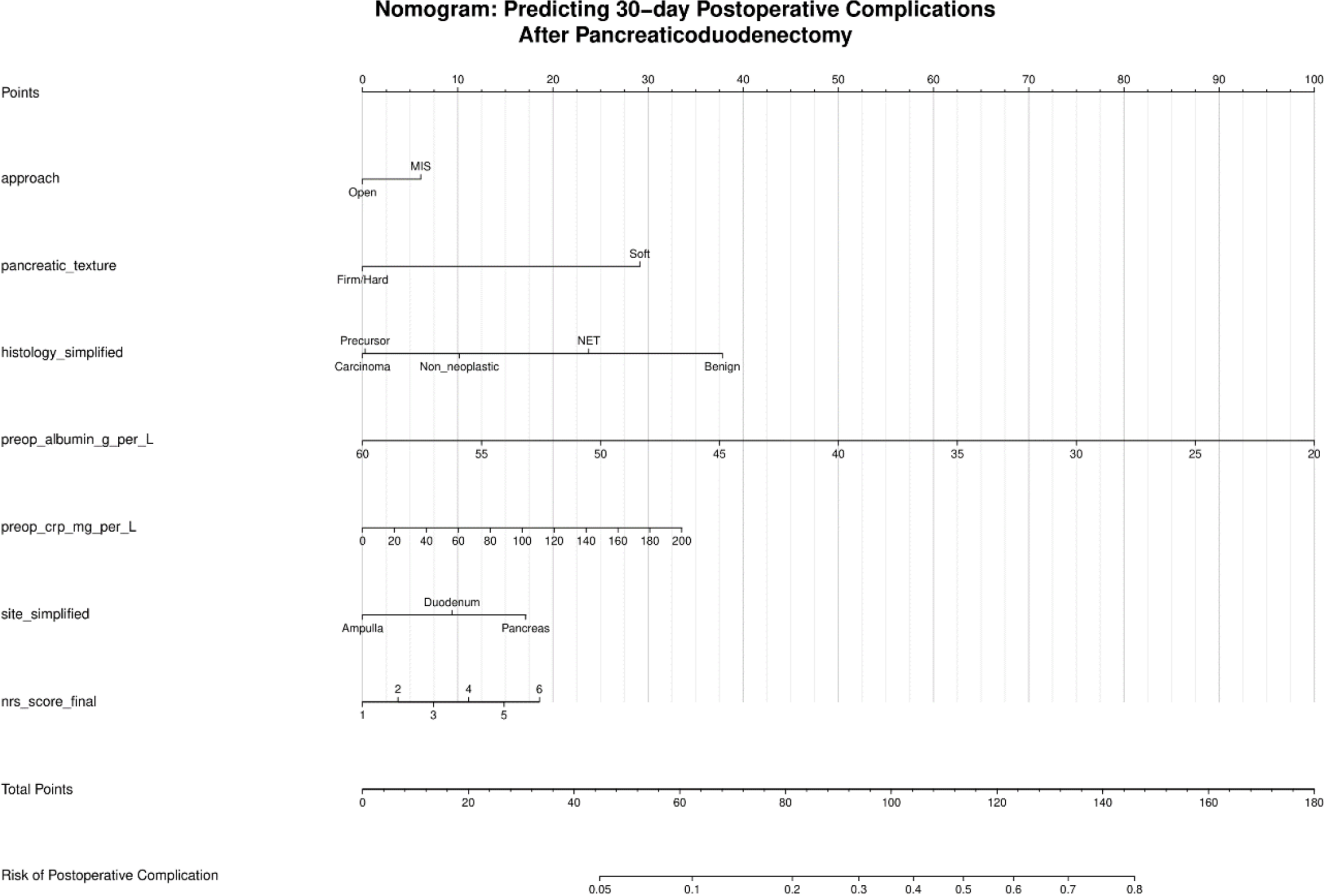
Nomogram for predicting the probability of any Clavien-Dindo grade II–IV complication within 30 days of pancreaticoduodenectomy. For each predictor, locate the corresponding value on its scale and draw a vertical line to the Points axis. Sum all individual point contributions to obtain Total Points, then project to the Risk of Postoperative Complication scale. Pancreatic texture (Soft vs Firm/Hard) is assessed intraoperatively. All remaining predictors — albumin (g/L), CRP (mg/L), histological diagnosis, anatomical site, NRS-2002 nutritional score, and surgical approach — are available from preoperative assessment.

The model achieved an apparent AUC of 0.747 (bootstrap 95% CI 0.704–0.825; **Table 6**, **Figure 2**). Bootstrap validation across 1000 iterations estimated a mean optimism of 0.043, yielding an optimism-corrected AUC of 0.704. Repeated 100-iteration stratified cross-validation produced a mean training AUC of 0.749 ± 0.015 and a mean validation AUC of 0.707 ± 0.063 (95% CI 0.597–0.841). The concordance between bootstrap-corrected (0.704) and cross-validation (0.707) estimates supports the stability of the performance assessment. Calibration was good (Hosmer–Lemeshow P = 0.686; **Figure 3**). The events per variable (EPV) was 13.9 (97 events across 7 predictors), meeting the recommended minimum threshold (25). The nomogram is presented in **Figure 4**.

**Table 6:** Model performance and internal validation metrics.

Metric	Value
*Total patients*	265
*Total events (Clavien-Dindo grade II–IV)*	97
*Event rate (%)*	36.6
*Final predictors (6 data-driven + 1 forced)*	7
*Nominal EPV*	13.9
*Effective EPV (dummy-variable count)*	8.1
*Effective parameters*	12
*Apparent AUC*	0.747
*Bootstrap 95% CI of apparent AUC*	0.704–0.825
*Optimism-corrected AUC*	0.704
*Bootstrap optimism*	0.043
*Repeated CV training AUC (mean ± SD, 100 iterations)*	0.749 ± 0.015
*Repeated CV validation AUC (mean ± SD, 100 iterations)*	0.707 ± 0.063
*Repeated CV validation 95% CI*	0.597–0.841
*Hosmer–Lemeshow P (g=8)*	0.686

AUC, area under the receiver operating characteristic curve; CI, confidence interval; CV, cross-validation; EPV, events per variable. The bootstrap 95% CI characterises uncertainty around the apparent AUC distribution across 1000 bootstrap samples. The optimism-corrected AUC (0.704) is a separate bias-corrected point estimate and is not the centre of this interval.

### Decision curve analysis

**Figure 5:**
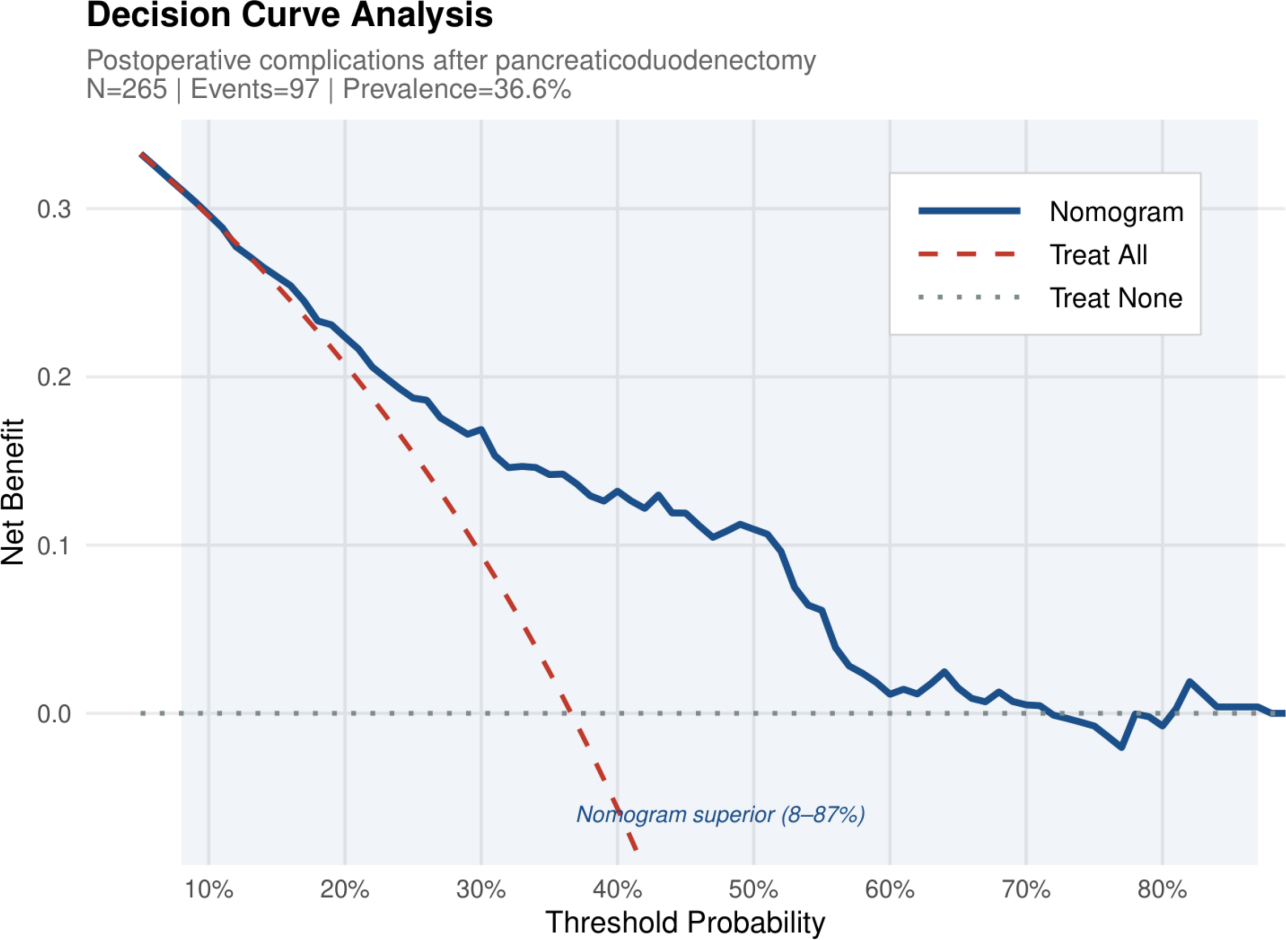
Decision curve analysis across threshold probabilities of 5–90%. The nomogram provides net benefit superior to both treat-all and treat-none strategies across approximately 8–87%. At a 30% threshold, nomogram net benefit is approximately 0.18 versus 0.10 for treat-all. N = 265; events=97; prevalence=36.6%.

Decision curve analysis demonstrated net benefit for the nomogram across threshold probabilities of approximately 8–87%, with superiority over both treat-all and treat-none strategies throughout this range (**Figure 5**; **Supplementary Table 3**). At a threshold of 30% — a clinically representative decision point at which perioperative management modification might be considered — nomogram net benefit was approximately 0.18 versus approximately 0.10 for treat-all and 0 for treat-none.

### Complication-specific performance

**Figure 6:**
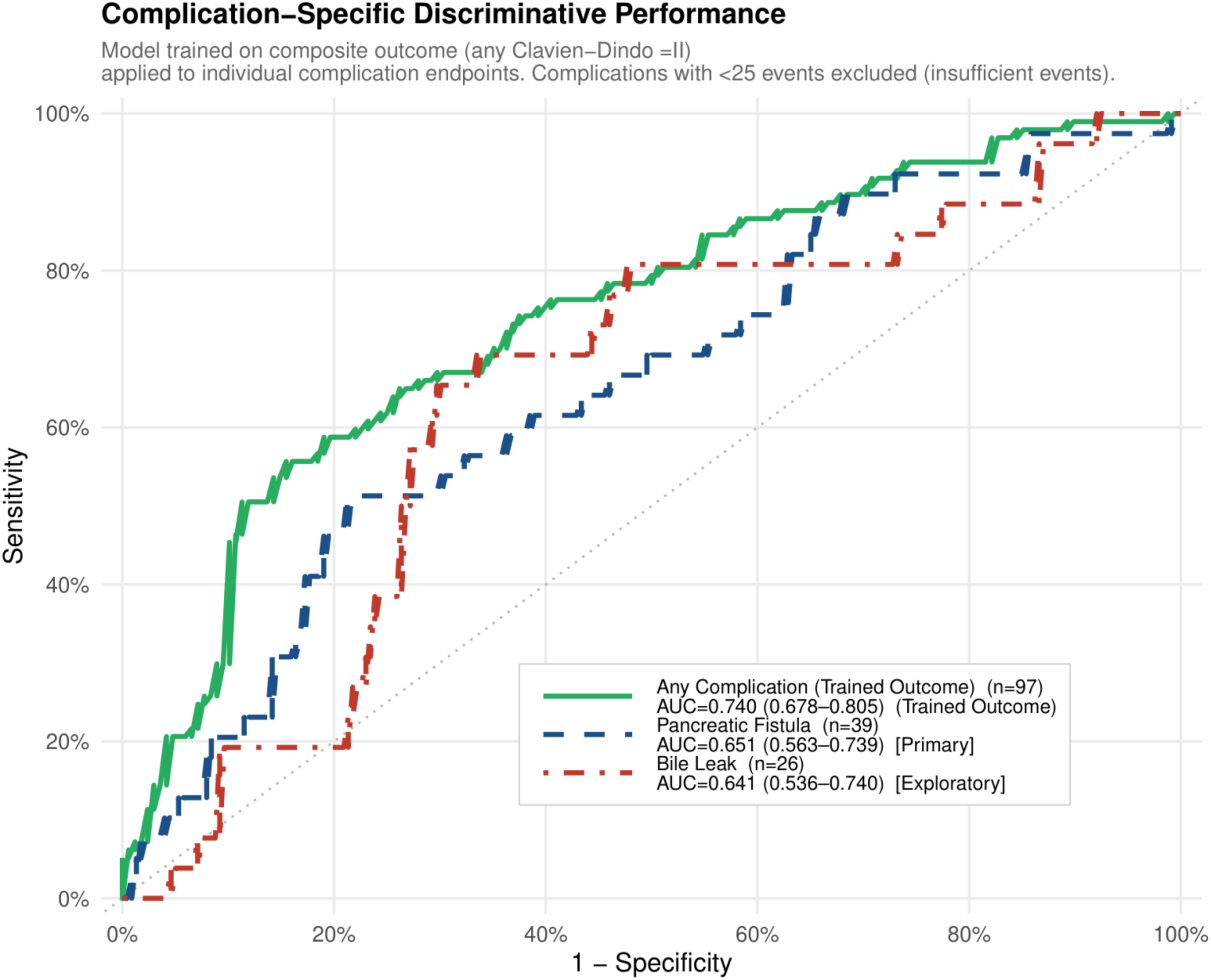
Complication-specific ROC curves. The composite-outcome model is applied *post hoc* to individual complication endpoints. The composite outcome AUC in this figure (0.740, 95% CI 0.678–0.805) uses DeLong standard CIs as displayed in the figure; the apparent AUC of 0.747 (bootstrap 95% CI 0.704–0.825) reported in the main text and **Table 6** uses 1000-iteration bootstrap resampling, which is the primary validation method. The minor difference reflects the two CI estimation approaches applied to the same ROC curve. Pancreatic fistula (n=39; AUC 0.651, 95% CI 0.563–0.739) and bile leak (n=26; AUC 0.641, 95% CI 0.536–0.740) are shown as primary and exploratory endpoints respectively. Endpoints with <25 events are not plotted; estimates are provided in **Supplementary Table 2**.

Applied *post hoc* to individual complication endpoints, the composite-outcome model yielded AUCs of 0.651 (95% CI 0.563–0.739) for pancreatic fistula and 0.641 (95% CI 0.536–0.740) for bile leak (**Figure 6**; **Supplementary Table 2**). Point estimates for intra-abdominal abscess (0.745, 95% CI 0.609–0.875), intestinal fistula (0.645, 95% CI 0.516–0.767), and delayed gastric emptying (0.745, 95% CI 0.607–0.863) are provided in **Supplementary Table 2**. Note that **Figure 5** displays the composite-outcome AUC as 0.740 (95% CI 0.678–0.805) using DeLong standard confidence intervals; the apparent AUC of 0.747 (bootstrap 95% CI 0.704–0.825) reported in the text and **Table 6** uses 1000-iteration bootstrap resampling, which is the primary validation method — the minor numerical difference reflects two CI estimation approaches applied to the same ROC curve. These modest complication-specific AUCs reflect the design intent of the model: trained on systemic physiological vulnerability, it is not optimised to discriminate individual complication subtypes governed by distinct local anatomical mechanisms.

### Subgroup analyses

**Figure 7:**
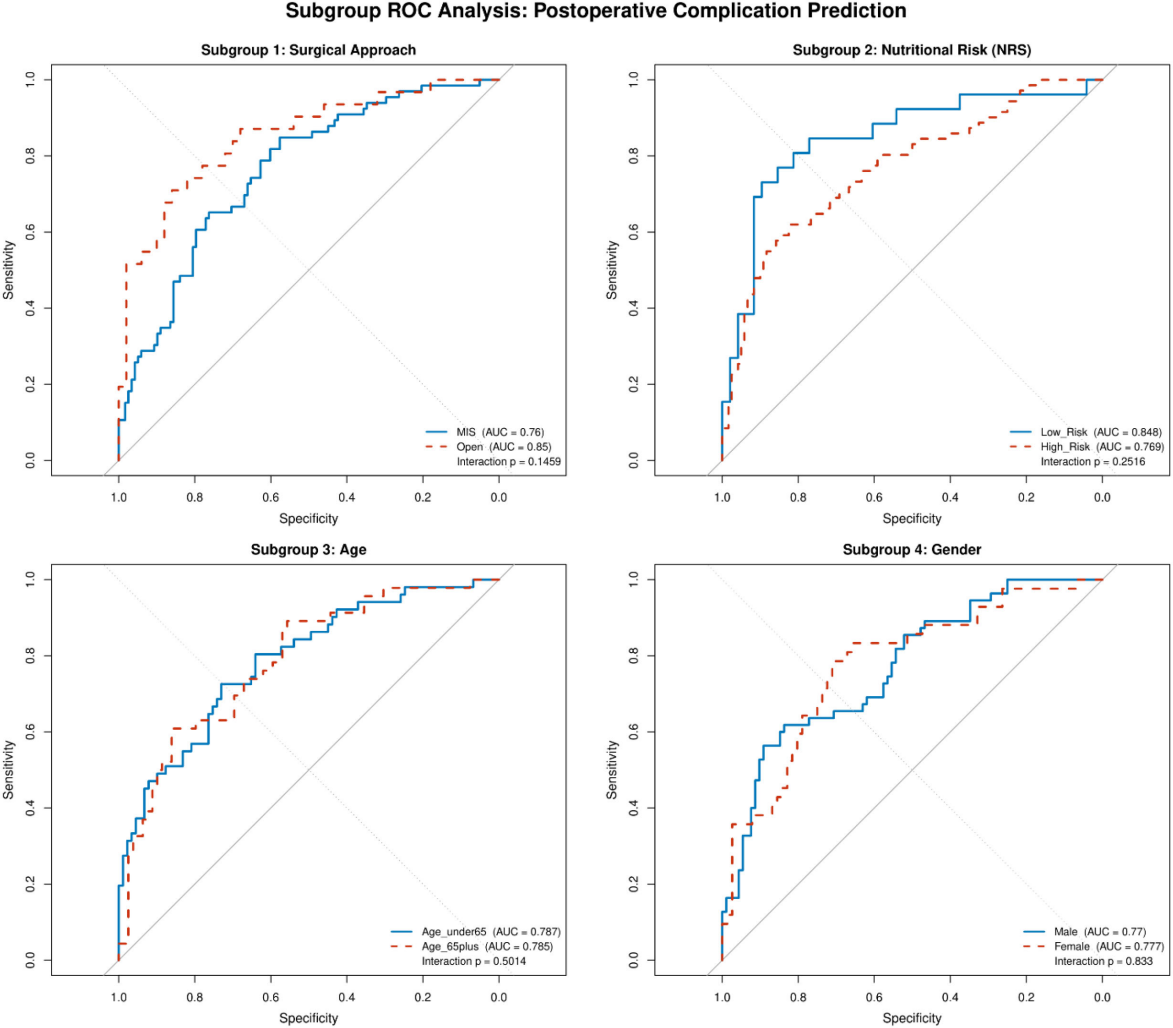
Subgroup ROC curves across four pre-specified strata: surgical approach (MIS vs Open), nutritional risk (NRS-2002 <3 vs ≥3), age (<65 vs ≥65 years), and sex. AUC values and likelihood ratio test P-values for interaction are shown. No statistically significant interactions were identified (all LRT P>0.05). Open subgroup AUC (0.850) based on 31 events; interpret with caution.

No statistically significant interactions were identified for surgical approach (LRT P = 0.146), nutritional risk (LRT P = 0.252), age (LRT P = 0.501), or sex (LRT P = 0.833; **Supplementary Table 1**; **Figure 7**). AUC was 0.760 in the MIS subgroup and 0.850 in the open subgroup, though the open estimate is based on 31 events in 81 patients and should be interpreted with caution. A noteworthy observation is the nominally poor calibration in the male subgroup (H-L P = 0.017), which, while not statistically confirmed as a systematic miscalibration, warrants prospective monitoring in external validation.

## Discussion

This study presents and internally validates a nomogram for predicting the full spectrum of clinically significant postoperative complications following pancreaticoduodenectomy, achieving an optimism-corrected AUC of 0.704 and good calibration across 265 consecutive patients with a 36.6% complication rate. The optimism-corrected AUC of 0.704 represents moderate discrimination, consistent with the performance ceiling inherent to a composite endpoint spanning five distinct complication subtypes with partially distinct aetiologies; this model is presented as an internally validated risk stratification instrument requiring external confirmation, and not as a definitive clinical tool. A notable finding of the data-driven variable selection process is the independent co-retention of two complementary nutritional predictors — serum albumin as a biochemical marker and NRS-2002 as a validated clinical nutritional risk tool — alongside intraoperative pancreatic texture, systemic inflammatory burden, pathological diagnosis, anatomical site, and surgical approach. While albumin has been incorporated in prior PD prediction models, and NRS-2002 has been evaluated as a predictor in broader abdominal surgery cohorts and within PD-specific nutritional nomograms (16, 26), the simultaneous data-driven co-retention of both markers within a single composite Clavien-Dindo morbidity model for PD appears not to have been previously reported, to our knowledge. This nutritional signal may reflect a gap in the pancreaticoduodenectomy prediction literature, which has predominantly focused on local anatomical parameters.

Preoperative hypoalbuminaemia was independently associated with postoperative complications in multivariable analysis (OR 0.925 per g/L; P = 0.016), a finding consistent with the established pathophysiological basis for this relationship. Albumin is a primary product of hepatic synthetic function; its reduction in states of protein-energy depletion may impair anastomotic healing through reduced availability of substrate for collagen synthesis and fibroblast proliferation, increase susceptibility to infectious complications, and compound the physiological stress of major surgery (11, 12). The observed reduction in albumin among complicated patients (35.79 versus 37.93 g/L; P = 0.001) is consistent with this association. The per-unit OR of 0.925 is broadly consistent with estimates reported in meta-analytic evidence from pancreatic resection cohorts (27), providing some external plausibility to the finding. Hypoalbuminaemia is a potentially modifiable preoperative risk factor: ESPEN guidelines recommend structured preoperative nutritional support for patients with a serum albumin below 30 g/L or with clinically significant nutritional risk prior to major abdominal surgery. (15) In this context, patients with a nomogram-predicted probability exceeding approximately 30% — at which threshold nomogram net benefit was 0.18 versus 0.10 for treat-all (**Supplementary Table 3**) — may be considered for: (1) structured preoperative nutritional optimisation per ESPEN guidelines; (2) enhanced perioperative monitoring; and (3) targeted preoperative risk discussion to inform consent.

The co-retention of NRS-2002 score as a complementary nutritional predictor alongside albumin reflects the multidimensional nature of preoperative nutritional risk in this population. While albumin captures the biochemical consequence of nutritional depletion — reduced hepatic synthetic output — NRS-2002 integrates the clinical composite of nutritional status impairment and disease-related increase in requirements, identifying patients at nutritional risk even before biochemical decompensation is manifest in the albumin level. The two predictors therefore represent non-redundant dimensions of the same underlying vulnerability: a patient with a normal albumin but a high NRS-2002 score has increased nutritional requirements that their intake cannot meet, placing them at prospective risk of depletion under the metabolic stress of surgery; conversely, a patient with hypoalbuminaemia may already have experienced biochemical depletion that NRS-2002 alone might not fully capture. The 72.1% prevalence of NRS-2002 ≥3 in this cohort — consistent with published nutritional risk rates in pancreatic and periampullary malignancy (13, 14) — highlights the frequency of preoperative nutritional vulnerability in this patient group. Both albumin and NRS-2002 are available from routine preoperative assessment without additional investigations, and their co-identification in this model may support the implementation of preoperative nutritional optimisation protocols in patients identified as high risk (15).

CRP was retained in the final model despite multivariable attenuation toward non-significance (P = 0.204), reflecting its strong univariate and LASSO signals and its biological role as the inverse acute-phase counterpart of albumin. In states of systemic inflammation — common in pancreatic and periampullary malignancy due to biliary obstruction, tumour-related inflammation, and nutritional depletion — CRP rises as albumin falls, with both markers capturing the same inflammatory-nutritional axis from opposing biochemical directions (28–30). Retaining both predictors allows the full range of this axis to be represented in the nomogram: albumin reflects the nutritional and inflammatory burden at the lower end, while CRP reflects acute inflammatory dysregulation that may not yet be manifest in the albumin level.

Pancreatic texture was the strongest predictor in this model (OR 3.490; P<0.001), consistent with its established dominance across both fistula-specific and composite complication prediction models (7, 8). The binary classification adopted here — Soft versus Firm/Hard — was pre-specified on mechanistic grounds: both Firm and Hard textures reflect parenchymal fibrosis conferring adequate suture-holding capacity and reduced exocrine secretion at the pancreatoenteric anastomosis, while the three-level classification lacks standardised inter-surgeon criteria for distinguishing Firm from Hard. The binary approach therefore reduces measurement imprecision without sacrificing biological validity. Pancreatic texture is assessed intraoperatively; consequently, while the majority of model predictors are available from preoperative assessment, the complete nomogram is applied at the perioperative stage once texture is known. This is acknowledged as a limitation of strictly preoperative applicability, and development of a strictly preoperative model excluding intraoperative variables is a priority for future work (31). Benign histological diagnosis was independently associated with significantly elevated complication risk (OR 4.734; P = 0.004). This association reflects the disease-biology relationship between pathological subtype and parenchymal physiology: benign and non-neoplastic lesions arise more frequently in soft, non-fibrotic glands, while pancreatic ductal adenocarcinoma is typically associated with upstream fibrosis and a firmer parenchyma. The histological signal therefore captures determinants of anastomotic behaviour that extend beyond the surgeon’s intraoperative texture assessment. This estimate carries a wide confidence interval given only 21 benign cases and requires external confirmation.

Surgical approach was included as a forced predictor on two pre-specified clinical grounds: the published evidence base demonstrating differential complication profiles between open and minimally invasive pancreaticoduodenectomy (17), and the predominance of MIS at this centre (69.4%). In settings where the majority of procedures are performed via one approach, surgical approach constitutes a potentially relevant source of outcome heterogeneity that may warrant adjustment in composite complication models, irrespective of its statistical signal in a dataset where approach distribution reflects case-mix rather than random allocation. The multivariable OR of 0.740 for open versus MIS (P = 0.364) is directionally consistent with outcomes data from predominantly MIS centres.

The variable selection sensitivity analysis (**Supplementary Table 4**) demonstrates that LASSO lambda.1se alone retained only three predictors (pancreatic texture, histological diagnosis, albumin), yielding an apparent AUC of 0.682 and an optimism-corrected AUC of 0.616 with acceptable calibration (H-L P = 0.540). The hybrid model retained seven predictors and achieved an apparent AUC of 0.747 and an optimism-corrected AUC of 0.704, representing a gain of +0.088 in corrected discrimination with equivalent calibration (H-L P = 0.686). The four additional predictors retained by the hybrid approach — anatomical site, NRS-2002, CRP, and surgical approach — contribute nutritional, inflammatory, and clinical-context information not captured by parenchymal and pathological variables alone. In a moderate-sized sample, LASSO at lambda.1se applies aggressive penalisation that may under-select predictors with genuine but moderate statistical signals; the hybrid approach addresses this by requiring convergent evidence across three methods before a variable is retained. The performance advantage of the hybrid approach nonetheless requires confirmation in external data.

Several limitations of this study require acknowledgement. The retrospective single-centre design introduces the potential for unmeasured confounding and limits generalisability to centres with different surgical volumes, technique distributions, and patient populations. Main pancreatic duct diameter — a key component of the Fistula Risk Score — was not prospectively collected, precluding direct benchmarking against fistula-specific prediction instruments. Pancreatic texture was assessed subjectively by the operating surgeon without standardised criteria, though the binary classification reduces the impact of inter-surgeon variability in gradating Firm versus Hard; formal inter-rater reliability assessment using kappa statistics was not performed in this retrospective cohort and is recommended for future prospective validation studies. The predominance of MIS (69.4%) may affect the generalisability of approach-related findings to centres with more balanced technique distributions. Complication-specific AUC estimates for endpoints with fewer than 25 events are exploratory and should not be over-interpreted. The male subgroup showed borderline calibration (H-L P = 0.017), warranting prospective monitoring in external validation. These limitations collectively reinforce that external, multi-centre, prospective validation is the necessary next step before clinical implementation.

## Conclusions

A seven-predictor nomogram incorporating binary pancreatic texture, histological diagnosis, anatomical site, CRP, surgical approach, serum albumin, and NRS-2002 score achieves internally validated perioperative risk stratification for clinically significant postoperative complications following pancreaticoduodenectomy, with a moderate optimism-corrected AUC of 0.704, good calibration, and positive net clinical benefit across threshold probabilities of approximately 8–87%. The data-driven co-selection of albumin and NRS-2002 as independent predictors — one biochemical, one clinical — was an exploratory finding of this analysis and suggests that preoperative nutritional assessment may warrant formal consideration in pancreaticoduodenectomy risk stratification pathways. In patients identified as high nutritional risk, structured preoperative optimisation represents a potentially modifiable approach to reducing postoperative morbidity, though this requires prospective evaluation. These findings are derived from a single-centre retrospective cohort, and external, multi-centre, prospective validation is required before clinical implementation.

## Data Availability

The raw data supporting the conclusions of this article will be made available by the authors, without undue reservation.
