# Supplementary material for: Development and validation of a nomogram for predicting postoperative complications after pancreaticoduodenectomy: a retrospective cohort study
Source: Front Oncol. 2026 May 20;16:1839358. doi: 10.3389/fonc.2026.1839358 (PMC13229620; doi:10.3389/fonc.2026.1839358)
Supplement: Supplementary file 1 [file DataSheet1.pdf]

Supplementary Tables

Development and Validation of a Nomogram for Predicting Postoperative Complications After Pancreaticoduodenectomy

Supplementary Table S1. Subgroup Analysis Results

Subgroup	Group	N	Events	Event %	AUC	H-L P	LRT P (interaction)
Surgical approach	MIS	184	66	35.9	0.760	0.900	0.146
	Open	81	31	38.3	0.850*	0.626	
Nutritional risk (NRS)	Low NRS (<3)	74	26	35.1	0.848	0.868	0.252
	High NRS (≥3)	191	71	37.2	0.769	0.165	
Age	<65	140	51	36.4	0.787	0.785	0.501
	≥65	125	46	36.8	0.785	0.575	
Sex	Male	147	55	37.4	0.770	0.017†	0.833
	Female	118	42	35.6	0.777	0.197	

*AUC, area under ROC curve; H-L P, Hosmer–Lemeshow P-value; LRT P, likelihood ratio test P-value for interaction; NRS, Nutritional Risk Screening 2002; MIS, minimally invasive surgery. No statistically significant interactions detected (all LRT P>0.05). †Male subgroup H-L P=0.017 warrants attention in external validation. *Open subgroup AUC based on 31 events in 81 patients; interpret with caution.*

Supplementary Table S2. Complication-Specific Discriminative Performance

Complication	N events	AUC (95% CI)
Any complication — trained outcome (Clavien-Dindo grade II–IV)	97	0.747 (0.704–0.825) apparent; corrected 0.704
Pancreatic fistula	39	0.651 (0.563–0.739) [Primary]
Bile leak	26	0.641 (0.536–0.740) [Exploratory]
Intra-abdominal abscess	17	0.745 (0.609–0.875) [Exploratory]
Intestinal fistula	14	0.645 (0.516–0.767) [Exploratory]
Delayed gastric emptying	11	0.745 (0.607–0.863) [Exploratory]

Model trained on composite outcome (any Clavien-Dindo grade II–IV) applied post hoc to individual complication endpoints. Bootstrap 95% CIs from 1000 iterations. Endpoints with <25 events are exploratory and should not be over-interpreted. The composite-outcome model is not optimised for individual complication-type discrimination; fistula-specific models incorporating main pancreatic duct diameter remain the standard for fistula prediction.

Supplementary Table S3. Decision Curve Analysis — Net Benefit at Key Thresholds

Threshold Probability	Nomogram Net Benefit	Treat-All Net Benefit	Treat-None Net Benefit
10%	0.296	0.296	0
20%	0.224	0.208	0
30%	~0.180	~0.100	0
40%	0.132	−0.057	0
50%	0.109	−0.268	0
70%	0.005	−1.113	0

80%	−0.008	−2.170	0

NB, net benefit. Values derived from the decision curve analysis (Figure 6). The nomogram becomes superior to treat-all at approximately 8% threshold probability. The 30% threshold is a clinically representative decision point at which perioperative management modification might be considered; at this threshold, nomogram net benefit is approximately 0.18 versus 0.10 for treat-all. N=265; events=97; prevalence=36.6%.

Supplementary Table S4. Sensitivity Analysis — Hybrid Model versus LASSO-Only Variable Selection

Method	N predictors retained	Apparent AUC	Optimism-corrected AUC	H-L P
Hybrid composite scoring (final model)	7	0.747	0.704	0.686
LASSO lambda.1se only	3	0.682	0.616	0.540
Difference (hybrid − LASSO)	+4 predictors	+0.065	+0.088	—

Variables retained in final model but not in LASSO-only: anatomical site, NRS-2002, CRP, surgical approach (forced).

LASSO lambda.1se alone selected three variables (pancreatic texture, histological diagnosis, albumin), yielding an apparent AUC of 0.682 and an optimism-corrected AUC of 0.616. The hybrid composite scoring model retained seven predictors and achieved an apparent AUC of 0.747, an optimism-corrected AUC of 0.704 (+0.088 over LASSO-only), and a cross-validation AUC of 0.707 ± 0.063. Both models showed acceptable calibration (H-L P>0.05). The four additional predictors (anatomical site, NRS-2002, CRP, and surgical approach) contribute nutritional, inflammatory, and clinical-context information not captured by parenchymal and pathological variables alone. External validation remains necessary. AUC, area under the receiver operating characteristic curve; H-L, Hosmer–Lemeshow.

Supplementary Table S5. VIF Screening Results — All Candidate Variables (N=265)

Variable	VIF / GVIF^(1/2df)
Pancreatic texture	1.09
Diabetes mellitus	1.08
Hypertension	1.16
CRP (mg/L)	1.16
Albumin (g/L)	1.14
NRS-2002 score	1.24
Sex	1.25
RBC ($\times 10^{12}$/L)	1.29
Age (years)	1.41
BMI (kg/m^2)	1.42
Operative duration (min)	1.44
Intraoperative transfusion	1.59
Blood loss (mL)	1.87
Total bilirubin (μmol/L)	1.21
AST (U/L)	4.45
ALT (U/L)	4.51
Histological diagnosis	1.32
Anatomical site	1.42
Surgical approach (MIS/Open)	1.37
Intraoperative infusion volume (mL)†	11.25
Intraoperative urine output (mL)†	10.24

VIF, variance inflation factor. GVIF^(1/2df) threshold applied: 10. Two variables — intraoperative infusion volume and intraoperative urine output — exceeded this threshold (VIF 11.25 and 10.24 respectively) and were excluded from candidate consideration due to multicollinearity prior to variable selection. †Excluded from candidate pool due to VIF >10 (multicollinearity), not due to missing data.

Supplementary Table S6. Missing Data Profile — All 33 Candidate Variables

Variable	Missing (n)	Missing (%)
Age (years)	0	0.0%
Sex	0	0.0%
BMI (kg/m²)	0	0.0%
Diabetes mellitus	0	0.0%
Hypertension	0	0.0%
NRS-2002 score	0	0.0%
Pancreatic texture (Soft vs Firm/Hard)	0	0.0%
Surgical approach (MIS / Open)	0	0.0%
Operative duration (min)	0	0.0%
Blood loss (mL)	0	0.0%
Intraoperative transfusion	0	0.0%
Anatomical site	0	0.0%
Total bilirubin (μmol/L)	0	0.0%
Histological diagnosis	1	0.4%
ALT (U/L)	1	0.4%
AST (U/L)	1	0.4%
CRP (mg/L)	2	0.7%
RBC (×10¹²/L)	3	1.1%
Albumin (g/L)	3	1.1%
Main pancreatic duct diameter (mm)	239	88.5%
D-dimer (μg/mL FEU)	117	43.3%
Procalcitonin — PCT (ng/mL)	131	48.5%
Alpha-fetoprotein (AFP)	138	51.1%
Preoperative amylase (U/L)	148	54.8%
Preoperative lipase (U/L)	148	54.8%
Carcinoembryonic antigen (CEA)	154	57.0%
Cancer antigen 125 (CA-125)	163	60.4%
Fasting insulin (μIU/mL)	171	63.3%
Carbohydrate antigen 242 (CA 242)	178	65.9%
Transferrin (g/L)	184	68.1%
Vitamin D — 25(OH)D (nmol/L)	196	72.6%
Retinol-binding protein (mg/L)	211	78.1%
Intraoperative infusion volume (mL)*	0	0.0%
Intraoperative urine output (mL)*	0	0.0%

*All predictors retained in the final model had ≤1.1% missing data (most 0%), justifying complete-case analysis throughout. Variables with >40% missing data were excluded from candidate consideration prior to variable selection. *These two variables had 0% missing*

data but were excluded from candidate consideration due to multicollinearity (VIF >10; see Supplementary Table S5), not due to missingness. This distinction is noted to avoid confusion regarding their absence from the variable selection process despite complete data availability.
